# Solutions to Peto's paradox revealed by mathematical modelling and cross-species cancer gene analysis

**DOI:** 10.1098/rstb.2014.0222

**Published:** 2015-07-19

**Authors:** Aleah F. Caulin, Trevor A. Graham, Li-San Wang, Carlo C. Maley

**Affiliations:** 1 Genomics and Computational Biology Graduate Program, University of Pennsylvania, Philadelphia, PA 19103, USA; 2 Department of Pathology and Laboratory Medicine, University of Pennsylvania, Philadelphia, PA 19103, USA; 3 Evolution and Cancer Laboratory, Barts Cancer Institute, Barts and the London School of Medicine and Dentistry, Queen Mary University of London, London EC1M 6BQ, UK; 4 Biodesign Institute, School of Life Sciences, Arizona State University, Tempe, AZ 85287, USA; 5 Center for Evolution and Cancer, University of California San Francisco, San Francisco, CA 94143, USA

**Keywords:** Peto's paradox, evolution, cancer, tumour suppression, algebraic model, Wright–Fisher model

## Abstract

Whales have 1000-fold more cells than humans and mice have 1000-fold fewer; however, cancer risk across species does not increase with the number of somatic cells and the lifespan of the organism. This observation is known as Peto's paradox. How much would evolution have to change the parameters of somatic evolution in order to equalize the cancer risk between species that differ by orders of magnitude in size? Analysis of previously published models of colorectal cancer suggests that a two- to three-fold decrease in the mutation rate or stem cell division rate is enough to reduce a whale's cancer risk to that of a human. Similarly, the addition of one to two required tumour-suppressor gene mutations would also be sufficient. We surveyed mammalian genomes and did not find a positive correlation of tumour-suppressor genes with increasing body mass and longevity. However, we found evidence of the amplification of *TP53* in elephants, *MAL* in horses and *FBXO31* in microbats, which might explain Peto's paradox in those species. Exploring parameters that evolution may have fine-tuned in large, long-lived organisms will help guide future experiments to reveal the underlying biology responsible for Peto's paradox and guide cancer prevention in humans.

## 1. Background

It is an open question why an elephant, with 100× more cells than a human, or a whale with 1000× more cells than a human, has approximately the same (or lower) cancer risk as a human [1]. This is Peto's paradox, and though many potential solutions have been proposed, it remains unsolved [2–5]. The fact that cancer rates are approximately constant across body sizes and lifespans suggests that there has been selection on the life histories of organisms to prevent cancer in large, long-lived organisms [2,3]. In order to investigate Peto's paradox, it would be helpful to understand how much evolution would have to change the parameters of somatic evolution to compensate for the evolution of large bodies and long lifespans. For example, we can ask how much the somatic mutation rate must decrease in order for a whale, which has 1000× more cells than a human, to retain the same cancer risk as a human.

**Figure 1.**
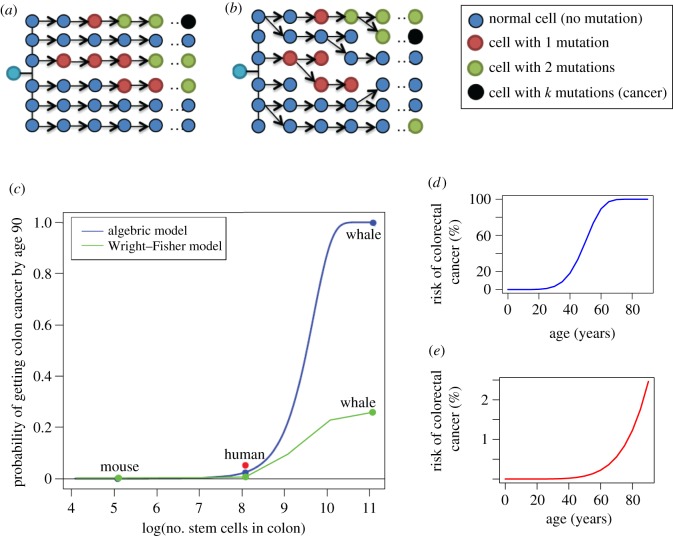
Estimated risk of colorectal cancer relative to body size under an algebraic and Wright–Fisher model. In the algebraic model (*a*) [7], cell lineages accumulate mutations over time, which are passed on to their daughter cell in the next generation and there is no cell death. In the Wright–Fisher model (*b*) [6], cells gain mutations over time, but each lineage has a chance of dying and being eliminated from the population. In both models, cancer occurs when a cell accumulates *k* mutations. The single light blue cell represents the zygote to show that all cells came from a single initial lineage. The probability was calculated using the algebraic and Wright–Fisher models with the parameters listed in table 1 [7] (*c*). Blue/green dots for mouse, human and whale indicate the estimated risk of colon cancer occurring within 90 years of life given the approximate number of cells in a human colon, 1000 times fewer cells to represent the mouse, and 1000 times more cells to represent the whale. The red dot indicates the lifetime risk of colon cancer according to the American Cancer Society which is about 5.3% for men and women averaged together [12]. The estimated age incidences of cancer for whale and human, given the algebraic model, are shown in (*d*) and (*e*), respectively. (*c*–*e*) Adapted from [2] with permission from Elsevier.

Computational models of cancer risk [3,6–10] can be used in comparative oncology to estimate how cancer risk should scale across body size and lifespan, and to test how much mutation rate, generation time, number of stem cells and the number of mutations would have to evolve to compensate for the evolution of large bodies and long lifespans. We used an algebraic model [7,11] and a Wright–Fisher model [6], which are generally similar, except that the Wright–Fisher model allows for cell lineage death (figure 1*a*,*b*). We then used the available genomic data for mammals to look for evidence that the number of tumour-suppressor genes may have increased to compensate for large bodies and long lifespans.

## 2. Results

### (a) Model 1: algebraic model of cancer incidence

Calabrese and Shibata [7] devised a simple mathematical equation to express the probability of a human developing colorectal cancer given their age that closely matches the SEER cancer incidence data [13]. The probability of an individual developing colorectal cancer after a given number of stem cell divisions is
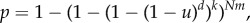
where *u* is the mutation rate per gene, per division; *d* is the number of stem cell divisions since birth; *k* is the number of rate-limiting mutations required for cancer to occur; *N* is the number of effective stem cells per crypt and *m* is the number of crypts per colon [7].

We varied the parameter *m* from 1.5 × 10^3^ to 1.5 × 10^10^ to see how the total number of stem cells in the colon changes the lifetime (90 year) risk of developing colorectal cancer (figure 1*b*). Estimates from human and mouse suggest that for every order of magnitude increase in body size, the number of crypts increases proportionally (see §4 Material and methods). Each crypt likely houses a similar number of stem cells so this corresponds to a proportional increase in stem cell number. Otherwise, we used the same parameter values as Calabrese and Shibata (table 1) to allow an easy comparison with their original results, though these are estimates and some measurements (e.g. stem cell division rates) are still under investigation.

**Table 1.** Model parameters. These parameters were used for the algebraic model to see how colorectal cancer incidence scales with body size. Parameter values were taken from [7]. The mutation rate assumes that there are three genes (1 kb each) per pathway and a background mutation rate of 10^−9^ mutations per base pair per cell division.

parameter	value	definition
*u*	3 × 10^−6^	mutations/oncogenic pathway/cell division
*d*	age(days)/4	divisions since birth (rate = 1 div./4 days)
*k*	6	rate liming mutations required for cancer
*N*	8	effective stem cells per crypt
*m*	(1.5 × 10^−3^–1.5 × 10^10^)	crypts per colon

If a blue whale has *m* = 1.5 × 10^10^ colonic crypts, this model predicts that all blue whales would have colorectal cancer by age 90 (figure 1*c*). More specifically, when we solve the equation for years 0–90, we find over 50% of blue whales would have colorectal cancer by age 50 and all would have colorectal cancer by age 80 (figure 1*d*). The estimate for an animal 1000 times smaller than a human (e.g. a mouse) is barely above zero even after 90 years. In reality, a mouse only lives a maximum of 4 years [14], so based on this equation they should never get colorectal cancer (figure 1*c*). The chance of an individual person getting colorectal cancer by age 90 is about 2.5% according to this model (figure 1*c*,*e*) and 5.3% as reported by the American Cancer Society [12]. It is implausible that 100% of blue whales actually get colorectal cancer by age 80. Though we do not know how often blue whales get colorectal cancer, they have been reported occasionally to have other cancers [15,16] and can live for over 100 years [14].

**Figure 2.**
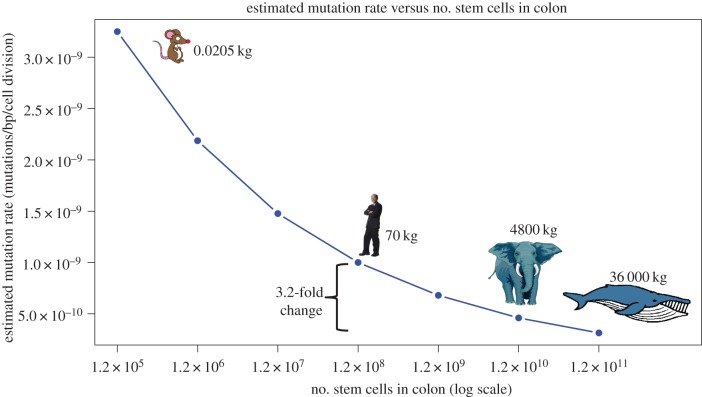
Estimated somatic mutation rates scaling with size. Mutation rate estimates show that a 3.2-fold decrease enables an animal that is 1000× larger (and so with 1000× more stem cells) than a human to have the same cancer risk. The mutation rates shown in the plot resulted in cancer risk predictions for the given number of cells that best matched the estimates for human (i.e. 1.2 × 10^8^ colonic stem cells) using the Calabrese–Shibata algebraic model [7].

Next, we investigated the set of parameter values that would allow the estimated age incidence of colorectal cancer in large animals to be similar to that of humans. We tested 10 000 mutation rates ranging from 3 × 10^−8^ to 3 × 10^−5^. A mere 3.2-fold decrease in mutation rate can account for a 1000-fold increase in body size (figure 2). The somatic mutation rates for an elephant and whale would need to be 4.6 × 10^−10^ and 3.13 × 10^−10^, respectively, in order for them to each have the same age incidence of colon cancer as humans (figure 2).

Additionally, we tested if altering the number of hits required for carcinogenesis (*k*) could allow cancer rates to be approximately equal across many orders of magnitude in size. We found that increasing the number of hits required for cancer was a powerful tumour suppressive mechanism. Keeping all other parameters consistent with the values listed in table 1, we varied *k* to range from 6 to 10. With 10 required hits, an animal 1000× larger than a human would have less than a 0.002% chance of getting cancer by age 90. However, just two extra hits (i.e. *k* = 8) for an animal this size gives the closest match to the human incidence curve (where *k* = 6) and is slightly below with a lifetime risk of only 1.5%.

Another hypothesis that has been proposed to explain Peto's paradox involves changing the dynamics, or population size, of the dividing stem cells in structures such as crypts. With this model, we find that even if each crypt contained only one stem cell, a whale would still be predicted to have a lifetime colorectal cancer risk of 96%, so this is an unlikely solution to the paradox. However, changing the stem cell division rate from once every 4 days to once every 13 days for an animal with one thousand times more crypts than a human reduces the lifetime cancer risk to 2.2% and the age incidence line closely matches that of human.

### (b) Model 2: Wright–Fisher model of cancer incidence

We next adapted a more realistic Wright–Fisher-based model of cancer initiation, which allows for cell lineage death [6]. We have simplified the model to maintain a constant population of size *N*, representing the crypt stem cells in the colon.

Using the same parameters (table 1) and calculating colorectal cancer risk across body sizes, we find that the Wright–Fisher model provides a much lower estimate of lifetime risk than the Calabrese–Shibata model. After 1000 simulations of a human colon, the 90-year cancer risk is only 0.4% and for 1000-times as many stem cells, representing a whale colon, just over 25% of individuals get colon cancer (figure 1*c*). These lower values are expected when using the same input as in the Calabrese–Shibata model because the incorporation of random cell lineage death lowers the probability of a cell becoming cancerous as it not only has to accumulate all *k* oncogenic mutations (these can also be thought of as *k* different pathways that must be disrupted in order to achieve a cancer phenotype), but it also must avoid being eliminated from the population. However, 25% is still an extremely high cancer rate when only considering one cancer type (i.e. colorectal cancer). In humans, the lifetime risk of most individual cancers are well below 10% with the exception of breast (12.4%) and prostate cancer (16.2%) [12].

We also note that the lifetime risk of colon cancer seems to level off around 25% for the largest species modelled (figure 1*c*). This inflection point is a consequence of the probability of losing a cell lineage becoming independent of population size when the population is sufficiently large in a Wright–Fisher model. The probability that a given cell in generation *t* has no progeny in generation *t* + *1* is equal to (1 − 1/*N*)^*N*^. As *N* increases, we can make the following approximation:
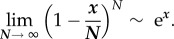
Therefore, when *N* is sufficiently large, the probability of cell lineage death is independent of the population size and becomes a constant (e^−1^ ≈ 0.37), which likely explains why cancer risk levels off when *N* ≥ 10^10^ with this model.

In this model, just one additional required hit for colon cancer (i.e. *k* = 7) can account for the risk due to the 1000-fold increase in cell numbers. This one additional hit, which represents the requirement for an extra pathway/gene to be disrupted in order to develop a cancerous phenotype, decreases the lifetime risk of large animals, like whales, to 0.6% which closely matches the human estimate of 0.4% for *k* = 6.

Decreasing the mutation rate for larger animals also greatly reduces their lifetime risk. Given 1.2 × 10^11^ crypt stem cells, a rate of 1.3 × 10^−6^ mutations (2.3-fold decrease) per oncogenic pathway per division decreases the lifetime risk of cancer to the same as humans. This result can also be obtained by decreasing the cell division rate to once every 8.5 days. This results in a lifetime risk of 0.5% and a rate of one division every 9 days lowers this below the human estimate to 0.2%.

### (c) Evolution of cancer gene families in mammalian genomes

**Figure 3.**
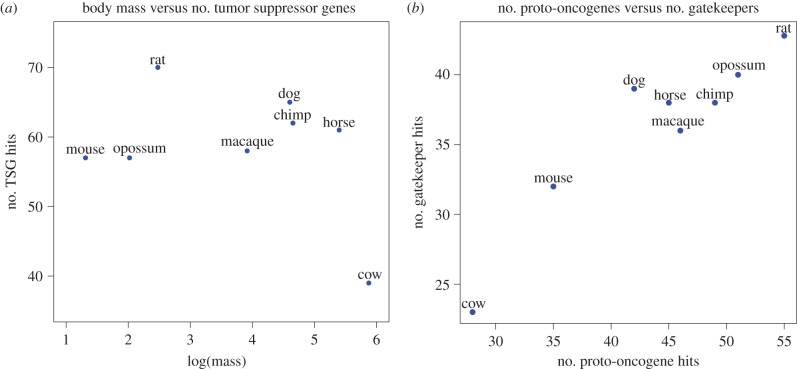
Cancer gene copy numbers across mammalian genomes. The number of tumour-suppressor genes does not increase with body mass (*a*). Based on our BLAST search, we find no positive correlation between tumour-suppressor genes as a whole, or GK and CT together with body mass. This was tested with a linear regression and is true on both the linear and log scale. The log (base 10) of the mass in grams is shown here to ease visualization of the range of masses. There is a strong linear correlation between the number of proto-oncogenes and GK (*b*). Based on our BLAST search for cancer gene families, the number of proto-oncogenes and GK found in a genome are highly correlated (*r*^2^ = 0.85, *p*-value < 0.001). Cow is the largest animal shown and has the lowest number of both gene types, though the rest of the data points are not in order of size.

It should be relatively easy for a species to evolve redundant checks on neoplastic progression by duplicating tumour-suppressor genes, which would present as expanded gene families in those species. Alternatively, a species could decrease the risk of progression by deleting proto-oncogenes [17]. We developed a genome-wide BLAST search intended to find all genes within a gene family based on one representative. We used the *TP53* gene family (*TP53*, *TP63* and *TP73*) as our positive control, with *TP53* as the query gene. In order for a BLAST hit to be considered as an instance of the given gene family, we required that it pass several filters based on coverage, significance, function and location (see §4 Material and methods). We applied the BLAST search and filters to a highly curated set of 81 cancer genes to count the number of proto-oncogenes and tumour-suppressor genes in eight mammalian genomes. The tumour-suppressor genes were further subdivided into ‘gatekeepers' (GK) and ‘caretakers' (CT) [18]. CT help maintain genome integrity by preventing DNA damage and performing DNA repair. These functions evolved billions of years before multi-cellularity and are essential to all forms of life [19]. GK control cell proliferation and signalling by enforcing checkpoints to ensure that cells at risk for neoplastic transformation do not continue to propagate. We did not find a positive correlation between body mass and the number of genome hits for any of the cancer gene categories (proto-oncogenes, GK and CT; tumour-suppressor gene results are shown in figure 3).

There is a weak negative correlation between body mass and the number of gatekeeper genes (*R*^2^ = 0.66, *p*-value = 0.015) or proto-oncogenes (*R*^2^ = 0.51, *p*-value = 0.047). The relationship is also true for the combination of GK and CT; however, CT alone do not show any significant correlation with mass (*R*^2^ = 0.37, *p*-value = 0.10) (figure 3*a*). These negative associations are driven solely by the lower counts found in cow and are completely abolished if the cow data-point is removed from the analysis. Interestingly, we found a strong correlation between the number of proto-oncogene and GK genes, which seems independent of size (*R*^2^ = 0.85, *p*-value < 0.001) (figure 3*b*). We do not find this relationship between proto-oncogenes and CT (*R*^2^ = 0.13, *p*-value = 0.36). There are no significant relationships between the number of genes in any of the cancer gene categories and lifespan, or the product of mass times lifespan.

### (d) Copy number of specific tumour-suppressor genes in mammals

Our BLAST analysis above is not sensitive enough to pick up small changes in individual gene copy numbers so we examined the copy number of specific tumour-suppressor genes in mammals. We focused on increased copies of tumour-suppressor genes as it is difficult to confirm a gene deletion in draft genomes due to possible incompleteness and mis-assemblies.

We used a comprehensive list of 830 human tumour-suppressor genes [20] and obtained the orthologous genes in 36 non-human mammals from EnsemblBioMart v. 72 (see electronic supplementary material, table S3). Genes that were found to have a ‘one : many’ relationship to the human tumour-suppressor gene in at least one mammal were considered for further analysis. Our results revealed that for 382 of the genes (46%), at least one species has one or more additional orthologues to the human gene, though often these are listed in the database as ‘apparent orthologues' and are not high confidence calls. Only 11% of the genes (99) have three or more paralogues in at least one mammal and this decreases to a set of 36 genes (4.3%) when we require a minimum of four copies of a gene. To limit false positives due to the unknown certainty of low copy number increases, we focused on the instances of extreme gene amplification. We found that 19 tumour-suppressor genes had five or more paralagous genes (i.e. at least four extra copies relative to the human genome) (table 2). Some genes in the list (e.g. *IL6* and *CTGF*) are perhaps better known for oncogenic activity; however, they are included in the list of 830 genes because there are published reports of them demonstrating tumour suppressive behaviour in certain tissues [20].

**Table 2.** Tumour-suppressor genes amplified in non-human mammals. This list includes all tumour-suppressor genes that we found to have at least four additional copies (i.e. five total copies) in mammalian genomes based on the ‘one: many’ orthologue annotation provided by Ensembl.

gene	common name	scientific name	copy no.
*FBXO31*	microbat	*Myotis lucifugus*	63
*TP53*	African elephant	*Loxodonta africana*	12
*IL6*	tree shrew	*Tupaia belangeri*	12
*LCN2*	guinea pig	*Cavia porcellus*	12
*CTGF*	lesser hedgehog tenrec	*Echinops telfairi*	9
*ING4*	rock hyrax	*Procavia capensis*	9
*ALOX15*	microbat	*M. lucifugus*	8
*MAL*	horse	*Equus caballus*	8
*MSMB*	opossum	*Monodelphis domestica*	8
	guinea pig	*C. porcellus*	6
*AKR1B10*	rat	*Rattus norvegicus*	7
*LIF*	rock hyrax	*P. capensis*	7
	African elephant	*L. africana*	5
*TCEB2*	rat	*R. norvegicus*	7
*TNFRSF 10A*	pig	*Sus scrofa*	7
*TNFRSF 10B*	pig	*S. scrofa*	7
*AKR1B1*	rat	*R. norvegicus*	6
*SLIT2*	cat	*Felis catus*	6
*CST5*	rat	*R. norvegicus*	5
*IFNB1*	cow	*Bos taurus*	5
	squirrel	*Ictidomys tridecemilineatus*	5
*S100A11*	bushbaby	*Otolemus garnettii*	5

Our results show a number of interesting outliers with evidence of massive gene amplification (table 2). The most extreme case is the *FBXO31* gene in the microbat (*Myotis lucifugus*) with 63 annotated copies. No other mammalian genome in the Ensembl database has more than one copy of this gene; however, the recent publication of the Brandt's bat (*Myotis brandtii*) genome reveals 57 copies of *FBXO31* [21]*.* This gene encodes an F-box protein that mediates the DNA damage response by promoting the degradation of cyclin D1 through polyubiquitination to induce cell cycle arrest in G1 [22]. Though the microbat is only 10 g, it can for live up to 34 years [14] so one hypothesis is that these additional tumour-suppressors may decrease the cancer risk of the bat, which would otherwise be heightened by their increased longevity [23].

The second highest gene copy number we came across was 12 which included *TP53*, *IL6* and *LCN2. TP53* is mutated in the majority of human cancers and plays a crucial role in multiple tumour suppressive pathways including apoptosis, senescence and DNA repair [24]. Redundant copies of this gene could greatly reduce the risk of tumorigenesis and has been experimentally shown in mice [25].

Additionally, the African elephant genome has five copies of *LIF* (leukaemia inhibitory factor). *LIF* is a target of p53 and can induce cell differentiation in immune cells [26]. However, the closest sequenced relative to the African elephant, the hyrax (*Procavia capensis*), has seven copies of *LIF.* When we looked at the mammals with less than five copies, we found that the lesser hedgehog tenrec (*Echinops telfairi*) also has three copies of the gene so we can assume that this amplification occurred before the divergence of these species within Afrotheria and, though it may be biologically interesting, it is not likely an explanation for Peto's paradox.

The other species listed in table 2 that are of interest include the horse (*Equus caballus*) and cow (*Bos taurus*)*.* The horse draft genome (*EquCab2*) has eight orthologues to the human tumour-suppressor gene *MAL*, which are located in tandem on scaffold 15*.* The only other species in the database with any duplicate copies is the microbat with a total of two *MAL* loci. This gene is involved in T-cell differentiation [27] and apical transport of membrane and secretory proteins [28–30]. Downregulation of this gene has been linked to multiple epithelial cancers, including colon, cervical and oesophageal [31–33]. The tumour suppressive properties of *MAL* have been verified in head and neck squamous cell carcinoma where the decrease of expression is associated with tumorigenesis, and the exogenous expression of *MAL* decreased cell proliferation and increased apoptosis [34].

The final gene from our analysis with more than four copies in a large organism is *IFNB1* found in the cow. This gene belongs to the class of interferon genes known for their role in triggering the immune response to eradicate pathogens and tumour cells [35,36]. However, we also see the same number of redundant copies (five) in the squirrel genome and two copies (i.e. one extra copy) in the guinea pig, horse and hyrax genomes, which makes it less likely to be directly involved with enhanced tumour suppression in large, long-lived animals.

## 3. Discussion

Essentially, all models are wrong, but some are useful.

—George E. P. Box [37, p. 424]

The algebraic and Wright–Fisher models used in this analysis are not intended to represent accurately the complexity of neoplastic progression; however, they are still useful for evaluating potential solutions to Peto's paradox. Interestingly, we find that the parameter changes that would be necessary to resolve Peto's paradox in large organisms fall within normal biological constraints. There is still much work to be done in the field to obtain more accurate estimates of human somatic mutation rates, as reported values span orders of magnitude and range from 10^−11^ to 10^−9^ mutations/base/division [38–42]. Though the estimates are not perfect, slight differences in mutation rate across species have been observed. For example, one study that derives somatic mutation rates from specific loci across eukaryotes found that the per base mutation rates for human and mouse are 5.0 × 10^−11^ and 1.8 × 10^−10^, respectively [39]. This is a 3.6-fold decrease in mutation rate in human versus mouse and is remarkably close to the results of our modelling, which suggest that a two- to threefold decrease in mutation rate can account for a 1000-fold difference in body size between mice and humans. This effective decrease in mutation rate may be accomplished by having better DNA repair in the larger species, more efficient removal of mutated cells, or less endogenous damage as a result of a lower mass-specific basal metabolic rate [2].

To decrease the lifetime risk of colon cancer sufficiently in large animals such as whales, we estimated that the stem cell division rate would only have to decrease from once every 4 days to once every 8.5 days, or 13 days, depending on the model. Crypt stem cells in mice divide once a day [43]; however, human measurements are limited and are estimated to be at least once per week [44,45]. One could investigate this by measuring the mitotic index of colonic crypts across species spanning orders of magnitude in size.

We were also able to resolve Peto's paradox by increasing the number of rate-limiting hits required for transformation. Both models show that with just one or two additional hits, the risk of cancer can be greatly reduced in large animals. Therefore, we might anticipate finding functionally redundant pathways or additional tumour-suppressor genes that act as a ‘back up’ in case of failure to existing pathways in animals that have evolved this tumour suppression mechanism better to combat cancer, which has been previously proposed as a solution to Peto's paradox [2,10,46]. The implications of this are not entirely straightforward though, as duplication of a tumour-suppressor gene could still have a dominant negative effect on the wild-type copies and would therefore not add the same type of protection as an independent gene with functional redundancy. However, our analysis of published animal genomes does not support our initial hypothesis that the total number of tumour-suppressor genes is increased in proportion to body mass.

Though we were able to use simple models to gain insight into a complicated disease, there are many assumptions that go into these models that we must acknowledge when interpreting the results. These models assume that all mutations are evolutionarily neutral for cell-level selection. The model also assumes a constant cell population size and mutation rate. Additionally, all *k* mutations necessary for cancer are required to occur in one single cell, which ignores the possibility of cell cooperation [47] and does not address clonal expansions, which would drastically alter the time to accumulate the mutations [48]. When oncogenic clones with a fitness advantage expand in the population, this greatly increases the chance that another oncogenic event will occur in a cell harbouring the prior mutation, which could decrease the time required for the evolution of malignant cells. Our model implementation does not consider fitness and selection of clones which limit its ability to realistically depict cancer; however, we can still gain theoretical insights and test hypotheses which could be pursued in the future with more appropriately detailed models and experiments.

Both models also assume that the rate-limiting step in carcinogenesis is the accumulation of oncogenic point mutations; however, other events can affect tumour initiation and progression. We do not address genetic changes involving chromosomal rearrangements, copy number changes, nor does this study consider epigenetic changes or alterations to the tissue microenvironment. This was done to gain an initial understanding of the parameters involved in carcinogenesis; however, all of the conditions and events listed above may be important *in vivo* and we are currently not capturing their contributions. Previous work has shown that within rodents, repression of telomerase activity (and therefore replicative senescence—an anti-cancer mechanism) coevolved with increased body mass, such that larger species have decreased expression [49]. This, along with other comparative studies revealing changes in expression in correlation with body size and/or lifespan [49–54], stresses the importance of continuing to expand our analysis of tumour suppression mechanisms in large long-lived organisms since evolution was not constrained to the set of parameters we have examined here.

The Wright–Fisher model (model 2) was originally developed to model one single crypt as it progresses from a benign polyp to an invasive tumour [6]. We have expanded the initial cell population to represent all stem cells in the colon; however, this ignores the compartmentalization structure provided by crypts, which are considered a barrier for the clonal expansion of pre-malignant cells [55,56]. This simplification to our model was made to reduce drastically the computational complexity and allowed for more direct comparisons with the Calabrese–Shibata model (model 1), which also did not consider the effects of the crypt structure.

A major caveat in this study is the difficulty in verifying a true gene deletion in a draft genome in the presence of incomplete assemblies, mis-assemblies and inaccurate annotations. There may also be undetected cancer genes in non-human species with little homology to the human gene sequences. However, we added the time since the most recent common ancestor with human to our linear model, to account for the difficulty of detecting genes with low levels of homology due to evolutionary distance, and this did not change the results, suggesting that the number of genes we find in each species is not simply a function of how closely they are related to humans. Human tumour-suppressor genes were used for this analysis, but in doing so we made the assumption that they perform the same function in the other species. This has not been experimentally verified. Additionally, we limited ourselves to these known tumour-suppressor genes, but there may be additional genes acting as tumour suppressors in other species that would have been missed, in addition to possible flaws in our filtering criteria that could cause some genes to be missed. As an example, we set the requirement that for two hits to be considered as separate instances of a query gene, they had to be at least 1 Mb apart; however, if a gene were duplicated in a tandem repeat, we would likely only count them as one copy.

Despite these limitations, we found genes that have been dramatically amplified in specific mammalian genomes, the most interesting of which is the discovery of 12 *TP53* copies in the genome of the African elephant. We subsequently cloned those genes and identified 19 distinct copies of *TP53* in African elephants and 15–20 in Asian elephants [1]. Another potential lead for solving Peto's paradox is *MAL*, which is found to have eight copies in the horse genome and two in microbat. This could be an example of convergent evolution where a large animal (horse) and a small, long-lived animal (microbat) both evolved extra copies of the same gene to overcome their increased risk of cancer. Further analysis and experimentation would need to be performed to determine the function of these copies and whether or not they provide enhanced suppression of carcinogenesis.

The goal of this analysis was to gain theoretical insight into the most realistic hypotheses to resolve Peto's paradox, rather than precise parameter estimations. We found that decreasing the mutation rate or division rate, or increasing the number of required mutations can all sufficiently reduce the lifetime cancer risk in an animal orders of magnitude larger than a human; however, decreasing the number of stem cells per crypt (or epithelial proliferative unit) is not a likely solution. The necessary changes in the mutation rates and number of required hits are small and are well within biologically feasible ranges. These values should be the focus of future experiments designed to measure the somatic mutation rates, stem cell generation times and the number of pathways that must be mutated to transform cells across species that span a wide range of sizes and lifespans. These data in the future may serve to identity the most effective strategies to prevent human cancer.

## 4. Material and methods

### (a) Justification for assuming that colon crypt count scales with body mass

A human colon is on average 1.5 m long and 6 cm in diameter [57], which gives an approximate area of 3 × 10^3^ cm^2^ (i.e. diameter × *π* × length). The total number of crypts is estimated to be 1.5 × 10^7^ [58,59], so the crypt density is approximately 5000 crypts per cm^2^. A mouse, which is three orders of magnitude smaller than a human, has roughly 6 cm^2^ of colon (6 cm long and 0.3 cm in diameter) [59]. Using the same crypt density, we calculate there to be approximately 3 × 10^4^ crypts in a mouse colon, which is the expected three orders of magnitude difference.

### (b) Calabrese–Shibata model

The Calabrese–Shibata model, which we have repurposed to explore solutions to Peto's paradox, was originally detailed in previous publications [7,11]. We use the same equation to calculate the risk of colorectal cancer given the age of the individual:
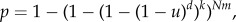
where *u* is the mutation rate per gene per division, *d* is the number of stem cell divisions since birth, *k* is the number of rate-limiting mutations required for cancer to occur, *N* is the number of effective stem cells per crypt and *m* is the number of crypts per colon [7]. We wrote a script in C (source code available upon request) to run through the model using ranges for each parameter and the results were plotted in R.

### (c) Wright–Fisher model

The Wright–Fisher model represents a constant population size, with non-overlapping generations, where each cell of the new generation choses a parent cell from which to inherit its mutant status. This occurs with equal probability (*1*/*N*) because we are not considering selective coefficients, to make it more comparable to the Calabrese–Shibata model and avoid using parameters that lack good experimental measurements. Given a population of *N* cells, the probability of a configuration of cells with 0 to *k* mutations at a given time (*t* + 1) can be expressed using the following multinomial distribution:
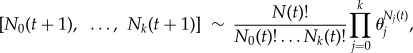
where *N*(*t*) is the size of the total population at time *t*, *N_j_*(*t*) is the population size of cells at time *t* with *j* mutations and *θ_j_* is the probability that a cell in generation *t* + 1 will have *j* mutations:
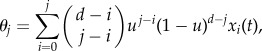
where *u* is the mutation rate per gene per generation, *d* is the number of potential driver genes and *x_i_*(*t*) is the fraction of cells with *i* mutations at time *t*. The number of potential driver genes *d* was set to six in this study to be comparable with the algebraic model having the parameters listed in table 1. This has been formally detailed in the original publication [6]. In our implementation, each instance of the model represents one colon with *N* crypt stem cells and all mutations are neutral. For each set of parameters, the model was run 1000 times to estimate the frequency of cancer. We ran a minimum of three independent replicates of the 1000 runs to make sure the number of cases reported to have cancer (i.e. contain *k* mutations) was consistent and we averaged across the replicates. R was used to visualize and plot the data.

### (d) BLAST analysis for gene family expansions

We retrieved protein sequences of more than 300 genes from the Cancer Genome Anatomy Project (CGAP) website [60]. We focused on genes with either oncogene (22 genes) or tumour-suppressor (59 genes) classification by CGAP (electronic supplementary material, table S1). Other genes were classified as partners of fusion genes by CGAP and were excluded from our analysis. We further divided the tumour-suppressor genes into two groups: CT (28 genes) if the gene had gene ontology annotations suggesting their functionality in DNA damage repair; otherwise genes were classified as GK (31 genes). We used the NCBI gene ontology annotation for human and checked for each gene whether it was associated with a gene ontology term (or a descendant of such term in the gene ontology hierarchy) having ‘DNA damage’ or ‘DNA repair’ in its description.

Genomes from the NCBI RefSeq database were used as BLAST databases against the 81 human cancer-related query genes to count the number of total hits in each genome. We limited the analysis to fully sequenced mammals at the time of analysis: cow, chimp, dog, horse, macaque, mouse, opossum and rat. For a BLAST hit to count as an independent instance of that gene in a given genome, it had to meet our criteria of coverage, significance, location, reciprocity and functionality. First, the union of all hits to that sequence in the subject's genome must cover at least 50% of the human query gene. Second, one of the BLAST hits in this region must have an *e*-value ≤ 10^−5^ and all other hits counting towards the 50% coverage must have *e*-values ≤ 10^−3^. Third, the BLAST hit must be greater than 1 Mb away from any other determined location of the query gene in the given subject genome. The location of hits for each organism, based on these criteria, was used as input into the UCSC genome browser to retrieve the predicted protein sequences determined by the N-SCAN algorithm. These sequences were then used for a reciprocal BLAST back to human RefSeq protein sequences (release 37). For a region to count as a true hit in a non-human species, the predicted protein sequence must return a top hit in the human genome that is either the original human query gene that produced that hit, or a paralogous gene. Paralogues were defined by the Ensembl genome browser (release 56). N-SCAN was also used to determine the functionality of the genomic regions to exclude known pseudogenes and intergenic regions that were not predicted to be genes. These criteria were determined by comparison of our results to known p53 gene families (as reported by Ensembl release 56) as a positive control. The numbers of hits for each of the 81 individual genes were tallied as proto-oncogenes, CT and GK for each organism.

Body mass data [14,61] and the evolutionary distance from humans were taken from the literature [62–66]. We fit a linear regression model to the data (electronic supplementary material, table S2) using the statistical package R to determine the relationship between the number of each gene type (proto-oncogenes, CT and GK) and the animal's body mass (representing the total number of cells in the organism). We tested this on both a log and linear scale.

### (e) Determining copy number of tumour-suppressor genes

A list of 830 tumour-suppressor genes was downloaded from the Memorial Sloan Kettering CancerGenes database [20] (for full list see electronic supplementary material, table S3). This list includes all genes that have been associated with tumour suppressive behaviour in at least one instance and have been assigned gene ontology terms related to these functions such as ‘positive regulation of apoptosis' and ‘negative regulation of cell proliferation’. Genes appear in this list regardless of whether or not they also have been reported to have oncogenic properties.

We obtained the orthologous relationships for 36 non-human mammals from Ensembl BioMart v. 72: alpaca (*Vicugna pacos*), armadillo (*Dasypus novemcinctus*), bushbaby (*Otolemur garnettii*), cat (*Felis catus*), chimpanzee (*Pan troglodytes*), common shrew (*Sorex araneus*), cow (*B. taurus*), dog (*Canis lupus familiaris*), dolphin (*Tursiops truncatus*), African elephant (*Loxodonta africana*), ferret (*Mustela putorius furo*), gibbon (*Nomascus leucogenys*), gorilla (*Gorilla gorilla gorilla*), guinea pig (*Cavia porcellus*), hedgehog (*Erinaceus europaeus*), horse (*E. caballus*), kangaroo rat (*Dipodomys ordii*), lesser hedgehog tenrec (*E. telfairi*), macaque (*Macaca mulatta*), marmoset (*Callithrix jacchus*), megabat (*Pteropus vampyrus*), microbat (*M. lucifugus*), mouse (*Mus musculus*), mouse lemur (*Microcebus murinus*), opossum (*Monodelphis domestica*), orangutan (*Pongo abelii*), panda (*Ailuropoda melanoleuca*), pig (*Sus scrofa*), rabbit (*Oryctolagus cuniculus*), rat (*Rattus norvegicus*), rock hyrax (*P. capensis*), sloth (*Choloepus hoffmanni*), squirrel (*Ictidomys tridecemlineatus*), tarsier (*Tarsius syrichta*), Tasmanian devil (*Sarcophilus harrisii*) and tree shrew (*Tupaia belangeri*). A phylogeny of the mammals used in this study is provided in electronic supplementary material, figure S1.

Genes that were found to have a ‘one : many’ relationship, as annotated by Ensembl, to the human tumour-suppressor gene in at least on mammal were considered for downstream analysis. The top genes were filtered based on the maximum number of times they occurred in any one species. All genes in table 2 occurred at least five times in the species indicated. The entire matrix of genes and copy number in each species is provided in the electronic supplementary material, table S3.

## Supplementary Material

Supplementary Table S3

## Supplementary Material

Supplementary Tables and Figures

## Data accessibility

The datasets supporting this article have been uploaded as part of the electronic supplementary material.
