# Supplementary material for: Solutions to Peto's paradox revealed by mathematical modelling and cross-species cancer gene analysis
Source: Philos Trans R Soc Lond B Biol Sci. 2015 Jul 19;370(1673):20140222. doi: 10.1098/rstb.2014.0222 (PMC4581027; doi:10.1098/rstb.2014.0222)
Supplement: Supplementary Tables and Figures [file rstb20140222supp2.pdf]

Electronic Supplementary Material

Table S1. List of Cancer Genes Used in BLAST Analysis
(22 oncogenes; 59 Tumor Suppressor Genes)

Gene_symbol	EntrezID	Classification
PTPN11	5781	proto-oncogene
AKT2	208	proto-oncogene
BCL2	596	proto-oncogene
BRAF	673	proto-oncogene
CDK4	1019	proto-oncogene
CTNNB1	1499	proto-oncogene
EGFR	1956	proto-oncogene
ERBB2	2064	proto-oncogene
FBXW7	55294	proto-oncogene
FGFR2	2263	proto-oncogene
GBAS	2631	proto-oncogene
HRAS	3265	proto-oncogene
KIT	3815	proto-oncogene
KRAS	3845	proto-oncogene
MET	4233	proto-oncogene
MYC	4609	proto-oncogene
MYCL1	4610	proto-oncogene
MYCN	4613	proto-oncogene
NRAS	4893	proto-oncogene
PIK3CA	5290	proto-oncogene
REL	5966	proto-oncogene
SMO	6608	proto-oncogene
APC	324	gatekeeper
BMPR1A	657	gatekeeper
CDH1	999	gatekeeper
CDKN2A	1029	gatekeeper
CEBPA	1050	gatekeeper
CYLD	1540	gatekeeper
EXT1	2131	gatekeeper
EXT2	2132	gatekeeper
FH	2271	gatekeeper
FLT3	2322	gatekeeper
FLT4	2324	gatekeeper
GATA1	2623	gatekeeper
GPC3	2719	gatekeeper
KLF6	1316	gatekeeper
MAP2K4	6416	gatekeeper
MEN1	4221	gatekeeper

10.1098/rstb.2014.0222

NF1	4763	gatekeeper
NF2	4771	gatekeeper
PHOX2B	8929	gatekeeper
PTEN	5728	gatekeeper
RB1	5925	gatekeeper
SBDS	51119	gatekeeper
SDHB	6390	gatekeeper
SDHC	6391	gatekeeper
SDHD	6392	gatekeeper
SMAD4	4089	gatekeeper
SMARCB1	6598	gatekeeper
STK11	6794	gatekeeper
SUFU	51684	gatekeeper
HNF1A	6927	gatekeeper
VHL	7428	gatekeeper
ATM	472	caretaker
BLM	641	caretaker
BRCA1	672	caretaker
BRCA2	675	caretaker
CHEK2	11200	caretaker
DDB2	1643	caretaker
ERCC2	2068	caretaker
ERCC3	2071	caretaker
ERCC4	2072	caretaker
ERCC5	2073	caretaker
FANCA	2175	caretaker
FANCC	2176	caretaker
FANCD2	2177	caretaker
FANCE	2178	caretaker
FANCF	2188	caretaker
FANCG	2189	caretaker
MLH1	4292	caretaker
MSH2	4436	caretaker
MSH6	2956	caretaker
MUTYH	4595	caretaker
NBN	4683	caretaker
PMS1	5378	caretaker
PMS2	5395	caretaker
RECQL4	9401	caretaker
TP53	7157	caretaker
WRN	7486	caretaker
XPA	7507	caretaker
XPC	7508	caretaker

10.1098/rstb.2014.0222

genus	species	mass(kg)	lifespan	ONC	CT	GK	MRCA
Macaca	mulatta	8.235	40	46	22	36	25
Pan	troglodytes	44.984	59.4	49	24	38	6
Canis	familiaris	40	24	42	26	39	97
Equus	caballus	250	57	45	23	38	97
Bos	taurus	750	20	28	16	23	97
Mus	musculus	0.0205	4	35	25	32	91
Rattus	norvegicus	0.3	5	55	27	43	91
Monodelphis	domestica	0.105	5.1	51	17	40	145

Table S2. Data used for linear model based on BLAST-analysis of cancer genes. Body mass data [1, 2] and the evolutionary distance from humans was taken from the literature [3-7]. ONC: oncogene; CT: caretaker; GK: gatekeeper; MRCA: most recent common ancestror with homo sapiens.

Table S3. Matrix of 830 Tumor Suppressor Genes used in Copy Number Analysis and the Number of Copies found in each of the 36 Mammals. Each row is a gene and each column is a mammalian species (see separate ESM file).

10.1098/rstb.2014.0222

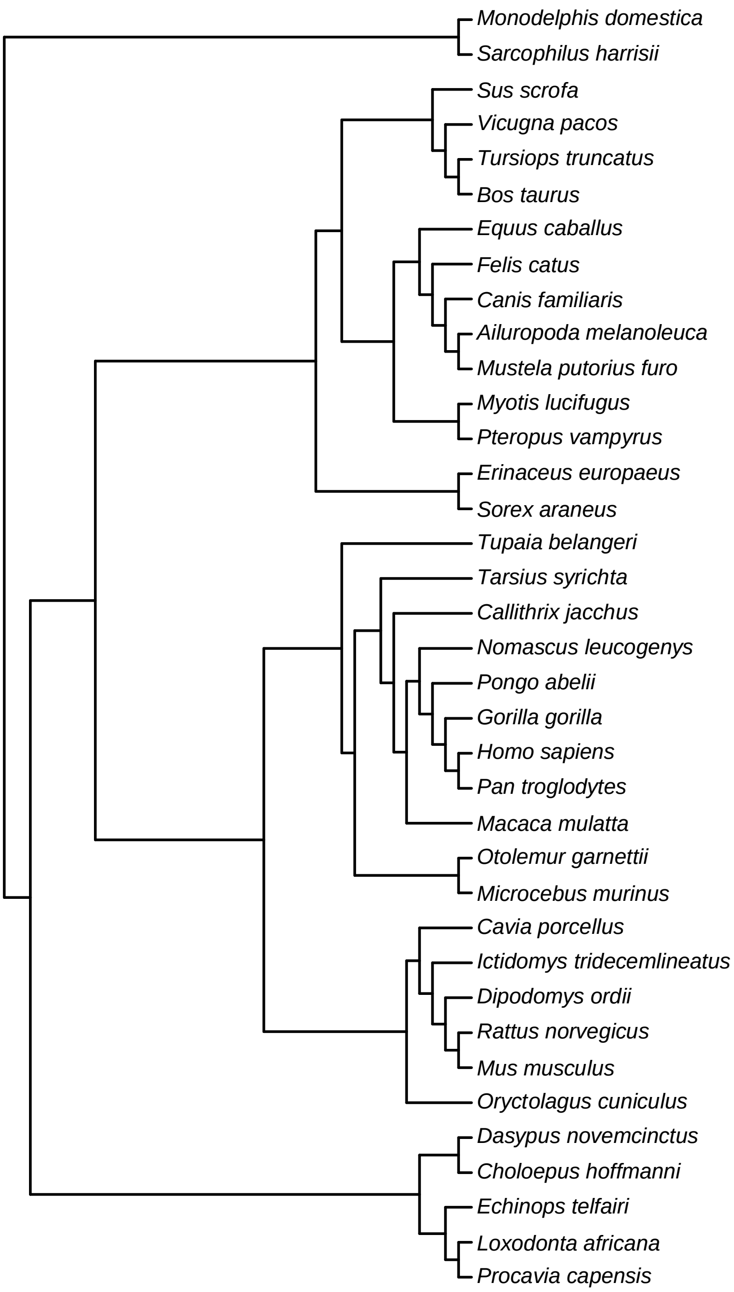

Figure S1. Phylogeny of 36 mammalian species used in the copy number of cancer genes analysis. The newick file of the phylogeny for all species in Ensembl was downloaded from Ensembl's gitHub repository (release 79) and plotted with the *ape* package in R. Species that were not used in this analysis but were in the Ensembl tree file were removed from the tree. *Homo sapiens* are shown for context.

10.1098/rstb.2014.0222

1. de Magalhães, J.P. and J. Costa, *A database of vertebrate longevity records and their relation to other life-history traits.* Journal of evolutionary biology, 2009. **22**(8): p. 1770-1774.
2. Smith, F.A., *Body mass of late Quaternary mammals*, in *Ecology*. 2003. p. 3403.
3. Bininda-Emonds, O.R., et al., *The delayed rise of present-day mammals.* Nature, 2007. **446**(7135): p. 507-12.
4. Chen, F.C. and W.H. Li, *Genomic divergences between humans and other hominoids and the effective population size of the common ancestor of humans and chimpanzees.* Am J Hum Genet, 2001. **68**(2): p. 444-56.
5. Gibbs, R.A., et al., *Evolutionary and biomedical insights from the rhesus macaque genome.* Science, 2007. **316**(5822): p. 222-34.
6. Murphy, W.J., et al., *Using genomic data to unravel the root of the placental mammal phylogeny.* Genome Res, 2007. **17**(4): p. 413-21.
7. Patterson, N., et al., *Genetic evidence for complex speciation of humans and chimpanzees.* Nature, 2006. **441**(7097): p. 1103-8.
